# Developing Youth Environmental Health Literacy and Civic Leadership through Community Air Monitoring in Imperial County, California

**DOI:** 10.3390/ijerph17051537

**Published:** 2020-02-27

**Authors:** Daniel Madrigal, Mariana Claustro, Michelle Wong, Esther Bejarano, Luis Olmedo, Paul English

**Affiliations:** 1 Tracking California, Public Health Institute, 555 12th St, Oakland, CA 94607, USA; michelle.wong@phi.org; 2 Comite Civico del Valle, 235 Main St, Brawley, CA 92227, USA; claustromariana@gmail.com (M.C.); esther@ccvhealth.org (E.B.); luis@ccvhealth.org (L.O.); 3 California Department of Public Health, 850 Marina Bay Pkwy P-3, Richmond, CA 94804, USA; paul.english@cdph.ca.gov

**Keywords:** environmental health literacy, youth leadership, community-based participatory research, community air monitoring

## Abstract

With a rapidly changing climate, new leaders must be trained to understand and act on emerging environmental threats. In California’s Imperial Valley, a collaborative of community members, researchers, and scientists developed a community air monitoring network to provide local residents with better air quality information. To expand the reach of the project and to prepare the next generation of youth leaders we developed an internship program to increase environmental health literacy and civic leadership. In the 10-week program, high school students learned about air quality science, respiratory health, community air monitoring, and policies intended to improve air quality. The students learned to present this information to their peers, neighbors, family, and community leaders. The program used participatory approaches familiar to community-engaged research to center the students’ experience. Surveys and interviews with the students were used to assess the program and found that the students became more familiar with air quality policies, increased their ability to use air monitoring resources, and increased their own confidence in their ability to effect change. With the growing threats related to environmental hazards, it is vital to prepare youth leaders to understand, communicate, and act.

## 1. Introduction

Climate change is rapidly affecting the environment with wildfires, extreme weather events, drought, and heat waves presenting new threats to human health [1]. The risks are complex and require new adaptation and mitigation strategies [2]. To enact these new strategies, a new generation of environmental health leaders will need to be prepared with the requisite knowledge and skills to attend to these challenges. We developed an internship program for high school students to develop environmental health literacy and civic leadership in California’s Imperial County, an area impacted by difficult environmental conditions including poor air quality. The internship was developed as a supplement to a citizen-science research project that installed a community air monitoring network in the area.

Imperial County sits along the US–Mexico border 90 miles east of San Diego. The population is mostly Latino (84.6% vs 39.5% in California) and many residents live in poverty (20.7% vs 12.8% in California) [3,4]. The county’s sources of air contaminants include cross border traffic, agricultural burning, recreational off-road vehicle use, and the desiccation of the Salton Sea, all of which contribute to the county’s high concentrations of particulate matter [5]. The county also has a high rate of asthma hospitalizations for children 5–17 years old [6]. In California, Latino residents are more likely to live in zip codes with the most environmental justice issues compared Californians on average [7]. Concerned residents were interested in learning about local air quality to better understand sources of air pollution and potential strategies to improve air quality.

To collect more local and usable air quality information, residents from the local advocacy group Comite Civico del Valle partnered with public health researchers of Tracking California (formerly the California Environmental Health Tracking Program) and air quality scientists from the University of Washington to create a network of 40 low-cost air monitors to measure particulate matter in real-time [8]. This citizen science project was developed with many of the principles of community-based participatory research, including prioritizing shared decision making, building capacity within the community, and providing new information that could be acted upon to improve air quality [9]. Community participation ensured the air monitors were sited in places prioritized by the community to increase the utility of the monitors [10]. The air monitor data was posted on a public website to provide residents with the ability to view particulate matter measurements at monitors near where they lived, worked, or attended school. The website also offered residents the option to sign up for alerts to announce when particulate levels were unhealthy, to let residents know they should avoid the air outside. Several monitors were placed at schools out of concern for children’s health. Comite Civico del Valle worked with school administrators to incorporate monitor data into school flag-programs to notify teachers, staff, students, and their families of unhealthy air quality with prominently displayed orange and red flags [10].

To increase the reach of the community air monitoring network, Comite Civico del Valle announced the project through the news media, social media, at community forums and other public gatherings. The project found a receptive audience with an environmental science high school class whose students connected the air monitor data to their course material. Recognizing the potential of youth leadership as a way to strengthen the resilience of the community to changing environmental conditions we developed a program to engage high school students. We developed the internship program to increase the understanding of the connection between health and the environment, and how to take action to effect change. The aim of this article is to evaluate the efficacy of the youth internship program in developing environmental health literacy and leadership as they relate to air pollution.

In this internship, we sought to increase environment health literacy among high school students by focusing on the interrelated topics of air quality, health, and the community air monitoring. Environmental health literacy is a growing area of interest within the field of environmental health research that examines the understanding of the connection between surroundings and health [11]. In the 2019 book “Environmental Health Literacy,” the first chapter defines the concept as “The basic knowledge and skills needed for comprehending environmental health risks and for devising, assessing, implementing and evaluating potential solutions [12].” There are several theoretical frameworks that have been proposed to develop environmental health literacy. In one of the first articles to describe environmental health literacy, Finn and O’Fallon proposed using Bloom’s taxonomy for its widespread use as a pedagogical framework and clear hierarchical structure [11]. According to Bloom’s taxonomy learning starts with simple engagement with concepts and gradually moves to more complex interactions with ideas. There are six phases of learning that follow the path of knowledge attainment: 1) remember, 2) understand, 3) apply, 4) analyze, 5) evaluate, 6) create. In another study focusing on developing environmental health literacy with high school students related to hormone disrupting chemicals in cosmetics, Bloom’s taxonomy was used to track the students’ progress in learning. In the beginning, the students were able to remember the concepts through memorization, then they interpreted the relationship of these product to their own lives, by the end the students were creating their own health education material for their peers. 

Another approach to develop environmental health literacy was described in the book “Environmental Health Literacy” in the chapter “Advancing Environmental Health Literacy through Community Engaged Research and Popular Education” [13]. In this chapter, three community-engaged research projects investigating environmental issues used the theoretical framework of popular education to center the lived experiences of the community members in the learning process. In each of these projects, the experiences of the community members were a starting point from which other new information was added. There was a deeper exploration of why the respective environmental hazards are present in their communities as they are, and most importantly the projects prioritized taking action to ameliorate the situations [13]. These steps were introduced as popular education by educator Paulo Freire in his work to teach adult literacy in Brazil [14]. We chose to use this theoretical framework with this internship because it was aligned with the orientation of the original community air monitoring research project that included substantial collaboration from both community members and researchers. As an extension of the spirit of the popular education approach we incorporated key principles of community-based participatory research: equitable decision making, emphasis on problems of relevance, and building capacity [9].

In addition to the development of environmental health literacy, we sought to develop leadership skills through the internship. After learning about local air quality issues, interns would share this information with peers and community leaders. By sharing information about air quality, health and community air monitoring they would deepen their own understanding and also would develop their own understanding of their own abilities to speak on topics they were concerned about. We recognize that the same benefits of youth participatory action research to increase engagement, develop a positive social identity, and for empowerment [15], could also be attained through a youth internship developed with participatory strategies.

## 2. Materials and Methods 

### 2.1. Methodological Design

The internship was created by Comite Civico del Valle and Tracking California, each contributing their knowledge related to environmental health research, health education, and working with high-school age youth. The design of this article is to evaluate the impact of the internship program and the participatory strategies employed. We examined the experiences of the students to assess the effectiveness of various elements of the internship. By conducting an evaluation of the researches we expect to reveal lessons learned and key practices that can be shared with other community-based participatory research programs interested in developing environmental health literacy and leadership with youth.

### 2.2. Description of the Internship Program

The internship curriculum was modeled after another community-based participatory research project conducted by UC Berkeley that incorporated the lived experiences of youth to amplify the learning of the connection between the environment and health. In the Health and Environmental Research in Make-up Of Salinas Adolescents (HERMOSA) study, youth researchers learned about endocrine disrupting chemicals in cosmetics while discussing their own experiences. They learned to share information on this topic with members of their community, and in the process developed a deeper understanding of the content and improved their own sense of agency [16]. In this internship, we adapted a similar model, with a focus on the air quality issues of Imperial County and the role of civic leadership in developing related policies. We also sought to develop the sense of agency and understanding reported in another youth driven participatory research project [17]. From these previous studies we concluded that there were various successful efforts in engaging youth in research projects that were dependent on creating a welcoming social environment and tailoring the program to the interests of the youth.

The internship curriculum was developed by staff at Tracking California and Comite Civico del Valle, incorporating the wide-ranging array of knowledge and experiences held by staff from these organizations. The internship was offered during the Fall of 2017, Spring of 2018, and Summer of 2018. The three cohorts had approximately ten high-school students and were led by two coordinators who were staff of Comite Civico del Valle. Staff of Tracking California and Comite Civico del Valle provided support and guidance in the administration and logistical planning of the internship. The internship recruitment was conducted by staff of Comite Civico del Valle to high school students, to families, to other community organizations, to school districts, and through informal conversations with community members. We recruited from youth attending high schools in Imperial County during the 2017–2018 academic year. The internship began with an application process that included an interview administered by the coordinators. We selected motivated students who demonstrated the capacity to actively participate in the internship. The interns, their families, the coordinators, and other staff of Comite Civico del Valle participated in an orientation meeting, where they learned about the purpose and activities of the internship. The interns participated in a series of 10 sessions with detailed agendas that were led by the coordinators (curriculum is available from the authors by request). The sessions were three hours long and were held weekly at a central location outside of school hours. Interns received $100 stipends for their participation in the internship. At the beginning of the three cohorts we started with team building activities to develop a supportive social environment where the students could develop trust within the group. Throughout the internship, the sessions were designed to allow interns to share their own stories of living in Imperial County while absorbing new scientific information related to air quality, layering more information each session.

The sessions of the internship were designed to emphasize the importance of the interns’ perspectives, the popular education approach. Each of the ten sessions began with activities where interns would discuss their own experiences living in the Imperial Valley. After, coordinators would present new information related to air quality, health, and air monitoring through hands-on activities, videos, lectures, and guest speakers. Next, the students were directed to reflect on how the new information related to their own experiences. The social bond within the group developed as interns shared these experiences, recognizing similarities and differences. The objectives and major activities of the ten sessions are described in Table 1.

In the first two sessions, the objective was to learn basic science related to air quality, health, and community air monitoring. The interns were instructed on how the community air monitoring network could be used to assess the health risks of various levels of particulate matter. With this information interns learned how to make an informed decision about whether to avoid the outside air by staying indoors. In the third session, the interns visited a ranger station near the Salton Sea to learn about how the drying up of this human-made lake could threaten air quality and human health. The Salton Sea visit demonstrated a tangible nearby impact of climate change. In the fourth, fifth, and sixth sessions, the interns learned how to give a semi-scripted ten-minute slide presentation on the community air monitoring network. During these sessions, the interns practiced their delivery and reflected on what was effective and what could be improved. After this preparation, each of the interns arranged and conducted at least four presentations on the community air monitoring network, either by themselves or in pairs, to audiences of their choosing. Interns reached out to their networks to set up these presentations to school classes, after school clubs, church groups, at local festivals, and at community forums. In these presentations, interns would demonstrate how to use the community air monitoring network website to learn about nearby particulate matter measures, and how this information could be used to avoid unhealthy air quality. At the seventh session, the interns learned about environmental law and regulations and learned about policies that were under consideration in the state legislature to improve air quality in the Imperial Valley. We presented information on the responsibilities of state and regional air quality boards to demonstrate their roles in monitoring and regulating air quality. We also explained the role of legislators in making state laws that affect emission reduction goals or mitigation and adaptation strategies. The presentation of civic structures was intended to inform the students of the systems in which decisions are made that impact policies that either allow or prevent environmental hazards.

In the eighth session they prepared for a visit to the state capitol in Sacramento, where they practiced delivering succinct messages in a fixed period of time to mimic a legislative office visit. The ninth session was a visit to Sacramento that included a tour of the capitol building, where they spoke to their elected representatives. In these meetings the interns discussed their experiences speaking with members of their community and their perspectives on current state bills related to environmental health. In the tenth session they reflected on their experience with the internship and discussed strategies to continue to be engaged in Imperial County air quality issues.

### 2.3. Evaluation Instruments

To evaluate the effectiveness of the internship in developing environmental health literacy and leadership, we developed a survey that was administered both at the first and last sessions of the internship. The survey included open questions on their motivation to participate in the program, attitudes towards topics discussed, and knowledge of air quality and regulation topics. The surveys included questions related to the knowledge, attitudes, and behaviors of the interns. Comparisons between percentage of agreement with statements related to environmental health literacy before and after the internship were assessed with a paired t-test in the statistical software program R [18]. After the last session, the interns participated in key informant semi-structured interviews that consisted of 10 questions. These interviews were recorded, transcribed, and reviewed to discover recurring themes and to collect detailed descriptions of the interns’ experiences. 

The State of California—Health and Human Services Agency’s Committee for the Protection of Human Subjects approved the youths’ participation in the assessment process and other research activities (protocol number # 17-01-2836) and informed consent was obtained from all youth.

## 3. Results

### 3.1. Implementation of the Internship

In the three cohorts of the internship, 29 interns participated in the sessions and completed pre and post surveys. The interns organized and conducted 103 presentations in Imperial County to 865 individuals. In these presentations, interns shared air quality and health information with community members and heard about the experiences of others living with poor air quality. Each of the cohorts participated in a visit to Sacramento that included a tour of the capitol building, meetings with elected representatives, and an official recognition of their visit by the legislature. Several enthusiastic interns were selected to give presentations at larger forums including the 2017 and 2018 Imperial Valley Environmental Justice Summits, and the 2018 Global Climate Summit. Interns also shared these same messages widely in their community through informal conversations with community members including peers, family members, neighbors, and elected representatives.

### 3.2. Perceptions of Environmental Health Literacy 

We evaluated the ability of the internship to develop environmental health literacy and leadership by assessing knowledge, attitudes, and behaviors related to air quality issues in Imperial County in pre and post surveys and an interview after the internship. Most environmental health literacy research focuses on the change in related knowledge, behaviors, and attitudes related to health outcomes and environmental hazards before and after the delivery of an educational program [19]. Our evaluation focused on the change in understanding of air quality issues and the interns’ ability to take action. To begin we assessed the reasons interns applied to the program, in the pre-internship survey we found that 41% joined to learn about air quality and 31% joined to make a difference. 

In the surveys, we compared the percent of interns who agreed with 16 statements before and after the internship. Table 2 shows the results of these changes. We saw relatively minor changes with the statements “I pay attention to the environment” (only an 11% increase), “I can make a real difference in improving my community” remained the same, and “I think science is important in everyday life” also remained the same. For the latter two, 82% of the interns responded in agreement with those two statements. For these statements, the interns started the program with a high degree of interest in environmental issues (75% agreement during pre-internship survey). The interns also came in with a high degree of agency in effecting change in their community and the importance of science. The high level of agreement at the beginning of the internship suggests the students were already engaged on the issues leaving limited space for improvement.

There was significant change in the number of interns in agreement with the statements “I know how to report an environmental concern to the government” (a 64 point increase), “I use air quality data to protect my health” (a 50 point increase), “I know how to plan a strategy to address community issues that are important to me” (a 57 point increase), and “I take action to address issues in my community that are important to me” (a 65 point increase). The changes in these statements demonstrate an increase in the interns’ understanding of the air quality regulation and monitoring system. Comparing the statements “I can make a real difference to improve my community” (which saw no change), to “I know how to plan a strategy to address community issues that are important to me” (which had a 57% point increase) and “I take action to address issues in my community that are important to me” (which had a 65% point increase) shows that while interns were aware of their agency, many had neither planned nor participated in an action that would benefit their community until this internship.

### 3.3. Impact of the Internship

In the interviews, when asked about the most useful parts of the internships, 45% of interns responded with the knowledge gained and 41% responded with the visit to the state capitol. One common response was that they did not expect to take on leadership tasks within such a short time. Several students reported expecting to learn about air quality science and respiratory health in a lecture format. Several interns reported being surprised once they realized that the work of the internship included speaking to community leaders. In Sacramento they shared what they learned through the sessions and from talking to community members about air quality issues in Imperial County. This visit was most impactful to 41% because they were able to see the impression their knowledge and experience made on their audiences. Legislative aides and elected representatives gave the students their full attention and valued their insights. One student answering the question which part of the internship was most valuable:

“One thing that surprised me was that a small group of kids from the Imperial Valley were able to talk to some of the most important people of Sacramento about the environment and about our flag system that we have here. And it kind of like shocked me that we have this kind of power even though we’re a very small county and very disproportionately Hispanic.”

Other students saw their visit to the decision makers in the capitol as an opportunity to express concern for issues that mattered to them. This student realized that he did not need to wait for an explicit invitation to discuss his opinion on issues that mattered to him:

“Part that was most valuable: To me, was the confidence to speak up when you see something that’s not right and you want to inform others because previously, in past years like I said, I wasn’t that too keen on speaking up.”

The interns also reported being positively affected by the people they interacted with throughout the internship, from their fellow interns, to the coordinators, to the guest speakers that shared expertise on a particular topic. As the interns became more comfortable with one another they recognized their shared interests:

“What I gained from the experience is building or creating friendships or relationships. Because as we were talking to the people, or in our little groups, since the more relationship we build off, the more comfortable we got to each other. Like speaking to the public really set our minds up into—how do I say this? It really enlightened us, ourselves, to express ourselves about air quality.”

While the internship presented the opportunities for growth, there were improvements that were suggested by the interns. The first recommendation from several interns in the post-internship survey was to extend the length of the internship to cover more material and to conduct more outreach. The interns felt the 10-sessions moved quickly and did not allow time for in-depth exploration of the topics. Another recommendation from the students was to have more hands-on activities during the sessions because they found these to be especially engaging and informative.

## 4. Discussion

The internship was designed to prepare youth to conduct peer and community outreach about air quality, health, monitoring, and regulation with the goal of increasing their environmental health literacy and civic leadership skills. The internship provided a structured experience where interns developed a deeper understanding of air quality, health, air monitoring, and their role in creating change. We did this by using the popular education approach and focusing on the experiences of the interns and emphasizing the importance of taking action to affect the problems they see. The development of the curriculum was informed by the community-based participatory research principles of: equitable decision making, emphasis on problems of relevance, and building capacity [9].

Through this evaluation of the internship we focused on the changes within the individual interns from the beginning to the end of the internship as it related to the specific environmental health focus: air quality, health, and community air monitoring. The challenge with any strategy that attempts to develop environmental health literacy is the wide breadth of knowledge and skills that are included in the concept. Gray and Lindsey describe five domains that comprise environmental health literacy: adult literacy, science literacy, health literacy, public health literacy, and environmental literacy [20]. Each domain covers an aspect related to the gathering of new information and each are required for a comprehensive understanding of the relationship between humans and their surroundings.

Despite the complexity of these overlapping domains, a recent review of the research found that most studies focus on a particular issue, rather than conduct a comprehensive assessment [19]. For example, a biomonitoring study near a Superfund site in Arizona found that returning results could be an effective way of increasing environmental health literacy and triggered health promoting behaviors as they related to contaminated waters [21]. Another study on exposure to endocrine disruptors through cosmetics in teenagers found that the participant-researchers increased their knowledge and their own confidence in their abilities to communicate scientific information through a community outreach program [16]. As these studies demonstrate, the changes in knowledge, attitudes, and behaviors are focused on the specific context, while contributing to an increase of a broader environmental health literacy.

As such we focus our analysis on the impact of the internship programs participatory methods (popular education and community-based participatory research). At the core of these approaches, the goal is to prioritize the experiences and perspectives of the subject. In this program, the subjects were the high school students living in Imperial County who participated in the internship. We examine the ways in which the participatory elements of the internship contributed to the development of environmental health literacy and leadership for the students.

### 4.1. Developing Environmental Health Literacy Through Participatory Methods

#### 4.1.1. Collaborative Partnerships

We developed the curriculum of the program drawing from the experience and knowledge from staff from Comite Civico del Valle and Tracking California. Staff at both organizations had experience creating curriculum to teach environmental health topics, and staff of Comite Civico del Valle worked in Imperial County to coordinate the logistics of carrying out the program. The internship coordinators worked with community leaders and local experts to set up visits during the sessions to provide examples to the students of local environmental health work in action. These individuals including other staff from Comite Civico del Valle maintaining the monitors, rangers at the Salton Sea overseeing climate change mitigation efforts, and legislative staff at the state capitol. The diverse set of collaborators contributed to the students experience by demonstrating a network of individuals working on the common goal of community empowerment and improvement. Each community leader that met with the students shared their own experiences working on environmental health issues on behalf of the valley, which provide new information for the students.

In addition, the role of the interns as key partners with decisions to make was essential for their development as leaders. They made decisions about what parts of their experience they would share and how they would present air quality issues in a manner that resonated with their peers and neighbors. The focus on equitable decision making emphasized the youths’ perspective throughout their internship experience. In Sacramento, it was the youth interns who decided which issues they felt should be discussed in their meetings with elected leaders. By emphasizing the intern’s decision-making power, they demonstrated their capacity to develop an opinion and share it with others. This was an important display of leadership both for themselves and for other residents of Imperial County.

#### 4.1.2. Emphasis on Public Health Problems of Local Relevance

Participatory research guided by community priorities are more likely to focus on relevant public health problems because people know what’s going on in their community [22]. The goal of improving community health attracted high school students to the internship and provided a compelling reason for audience members to hear the presentations of the interns. Many students were motivated by the prospect of making a positive impact in their community: making a difference was the reason 31% of high school students initially applied to the program. The interns grew up in Imperial County surrounded by poor air quality and high rates of childhood asthma. The concern becomes more urgent when the problems are nearby. By focusing on problems that were widely recognized as relevant in the Imperial County, interns were received by attentive community members interested in hearing about how the community air monitoring network could inform their understanding of local environmental conditions.

#### 4.1.3. Building Capacity 

The focus of the internship was to develop young environmental health leaders by providing skills and knowledge that will be useful to understand air quality issues, setting the framework to understand other environmental health issues. In the survey, four statements related to the interns engagement on environmental issues increase by over 50% over the course of the internship: “I know how to report an environmental concern to the government”, “I use air quality data to protect my health”, “I know how to plan a strategy to address community issues that are important to me”, and “I take action to address issues in my community that are important to me.” They demonstrate a profound shift in how the interns see their own agency. In addition to the knowledge and leadership skills they gained, the youth members learned about civics and how air quality related decision-making occurs in California. Part of that process was to develop professional relationships with community leaders that may be sources of future opportunities for interns. Each intern was guided through a process where they practiced their presentations until they felt comfortable enough to give them to a larger audience. Through repetition and supportive feedback, interns improved their presentation and public speaking skills. The interns improved their knowledge and abilities through the internship because the programming started with their own experiences. By ending the sessions with a visit to Sacramento, the internship highlighted the final steps of the popular education approach is to take action to improve the situation examined.

### 4.2. Lessons Learned

In the three cohorts of the internship, we prepared motivated high school students to give presentations on air quality issues. Through this experience we encountered the following lessons:

1. Focus on one topic. Each of the three internship cohorts lasted eleven weeks (orientation and ten weekly sessions), meaning that the reflecting, learning, and action planning was completed in under three months. We designed each session to meet multiple objectives related to team building, experience sharing, knowledge acquiring, and practicing essential skills like public speaking. The quick pace of the project was felt by interns who requested a longer internship length in the post-internship surveys.
2. Center the participant experience. In the popular educational framework, learning starts with the participants’ experience. Similarly, any educational program must be tailored to fit the needs and interests of the learner. In the internship, the participants were high school students with busy lives that sometimes conflicted with the internship activities. We had to be aware of the students’ schedule to know when they were available to meet. In addition, we also had to develop a curriculum that would provide air quality information in a manner that was engaging and informative.
3. Building relationships is key. The interns and coordinators built relationships with one another throughout the program. Each session both interns and coordinators shared something about their lives that allowed them to connect more deeply. The interns formed relationships among themselves, with program staff, and with other concerned community members. Additionally, it took time to prepare the sessions for the interns and to coordinate the schedules of the guest speakers. Because of these efforts, the interns left the internship with a larger network of people they know that are working on environmental health issues in their community. Following the program, interns from the three cohorts requested letters of recommendation from coordinators recognizing the value of staying connected with the program.
4. Encouragement eases self-doubt. One common concern expressed by the interns was their ability to present to an audience of their peers and to community leaders. We responded to this concern by building in exercises throughout the internship that built their confidence in their abilities and provided gentle feedback that allowed them to improve.

The lessons learned describe strategies that can be employed to realize the benefits of using popular education. The youth internship program’s primary objectives were to develop the students’ understanding of particulate matter, health, air monitoring and regulation, and their role in creating change. To achieve these objectives, we created an internship that attended to the students interests and concerns. We focused on the experience of the interns as a way to welcome them into the program, as a way to connect with what they know, and to assert the value of their leadership.

## 5. Conclusions

In the youth internship, high-school students shared their experiences growing up in Imperial County, learned about air quality and its relation to health, and shared this information within their community. While the students came in motivated to protect the environment and health, they were able to learn about the science behind air quality and health and about the system in place to protect human health from the worst impacts of poor air quality. Learning with the principles of popular education helped interns discover their own relationship to the environmental health topics discussed. By sharing this information with their peers, they attained a deeper understanding of the sources of particulate matter, how these pollutants are monitored and regulated, and their role in minimizing exposure to pollutants.

A participatory approach can be helpful in assessing environmental hazards and identifying promising mitigation strategies. The solutions of the future require the informed participation of young people, especially from the communities bearing the largest environmental burdens. These solutions can be discovered through thoughtful programming to build community, knowledge, and agency through approaches that center the lived experience of those affected. With the youth internship, we delivered one such model that expanded interns’ understanding of environmental health literacy and their own agency by focusing on their own experiences and intrinsic capacity for leadership.

## Figures and Tables

**Table 1:** Youth environmental health internship session titles, objectives, and activities.

Session Title	Objectives	Activities
Session 1. Intro to Air Quality and Health I	Learn about air quality science and respiratory health	Team building exercises and discussion about experiences living in the Imperial Valley followed by lectures and video.
Session 2. Intro to Air Quality and Health II	Learn about air quality science and respiratory health	Exercises to demonstrate the sources of air pollution, more lecture and video
Session 3: Visit to the Salton Sea	Learn about a local environmental hazard	Visit to the Salton Sea Ranger station
Session 4: The Community Air Monitoring Network Presentation	Introduction to the basic community air monitoring network presentation	Receive a detailed presentation on the community air monitoring network and how the various elements work together to teach
Session 5: Practicing the Community Air Monitoring network Presentation I	Build confidence in delivering the community air monitoring network presentation	Practice delivering the community air monitoring network presentation within the group
Session 6: Practicing the Community Air Monitoring Network II	Practice presentation community air monitoring network and make plans	Continue practicing the community air monitoring network presentation and making plans to give the presentation in the community
Session 7: Air Quality Laws and Regulations	Learn about air quality laws	Presentation on environmental policy making from an environmental lawyer along with a discussion period.
Session 8: Prepare to visit State Capitol	Prepare to speak with state representatives	Prepare for meeting with elected representatives
Session 9: Visit to State Capitol	Speak with state representatives	Visit the state capitol, take a tour of the capitol building, visit a legislative session, meet with elected representatives in their offices
Session 10: Reflection	Reflect on experience and consider what comes next	Reflection exercises where youth discuss their achievements throughout the internship and discuss strategies to continue to stay involved in the work

**Table 2:** Attitudes and behaviors of related to environmental health literacy and leadership before and after the youth environmental health internship.

	Percent Agree With Statement Before (n = 29)	Percent Agree With Statement After (n = 29)	Paired t-Test p-Value
I pay attention to the environment	75%	86%	0.2641
I know how to report an environmental concern to the government	25%	89%	< 0.001
I report environmental concerns to the government	4%	32%	0.001
I pay attention to the air quality	54%	86%	0.010
I know how to find out about air quality in Imperial County	64%	93%	0.005
I use air quality data to protect my health	21%	71%	< 0.001
I pay attention to problems in my community	57%	89%	< 0.001
I can make a real difference in improving my community	82%	82%	1.000
I know how to plan a strategy to address community issues that are important to me	11%	68%	< 0.001
I take action to address issues in my community that are important to me	14%	79%	< 0.001
I know how to present information to others about issues that are important to me	61%	89%	0.005
I communicate with peers about issues that are important to me	68%	86%	0.023
I communicate with elected officials and other leaders about issues that are important to me	11%	57%	< 0.001
I think science is important in everyday life	82%	82%	1.000
